# Topic: Continuous Enzymatic Peracids Synthesis in Pickering Emulsions: Influence of Nanoparticles Modification, Aqueous Phase Composition and Operational Parameters

**DOI:** 10.1002/elsc.70077

**Published:** 2026-04-07

**Authors:** Lemuel Onoriode Adomi, Sara Fatima Bhutta, Marion B. Ansorge‐Schumacher, Anja Drews

**Affiliations:** ^1^ School of Life Science Engineering HTW Berlin – University of Applied Sciences Berlin Germany; ^2^ Institute of Microbiology Technische Chair of Molecular Biotechnology Universität Dresden Dresden Germany

**Keywords:** *Candida antarctica* Lipase B, enzyme deactivation, membrane reactor, peroxyacetic acids, Pickering emulsions

## Abstract

The lipase‐catalyzed oxidative functionalization of alkenes in Pickering emulsions (PE) offers a green and efficient alternative to conventional processes involving hazardous oxidants. Other investigated reaction media used for this alternative green pathway still suffer from enzyme deactivation and /or low specific reaction rate which limits their industrial adaptability. This study investigates the enzymatic synthesis of peroxyacetic acid, the first step in lipase‐catalyzed oxidative functionalization, in a continuous membrane reactor. The effects of aqueous phase composition, modified silica nanoparticles, and operational parameters on reaction performance were systematically studied. Optimal conditions were obtained at pH 7, 100 mM buffer, and 5 g L_dp_^−^^1^ enzyme concentration. Surface‐modified silica nanoparticles improved PE stability and interfacial catalytic efficiency, while maintaining comparable catalytic productivity. Hydrogen peroxide outperformed urea hydrogen peroxide, yielding a maximum product yield of 83% at a concentration of 45 mM in the influent solution and a space‐time yield of 44.9 g_PA_L^−^^1^d^−^^1^ at 386 mM. At 386 mM, the specific reaction rate (17.5 mmol g^−^^1^ h^−^^1^) was over twice that of reported single organic‐phase systems. Despite the high peroxide concentrations, the enzyme displayed remarkable stability in PE due to the protective role of nanoparticles. This work provides critical insights into optimizing enzymatic oxidative functionalization in PE and their potential for sustainable industrial applications.

*Practical application:* This study provides a foundation for the development of sustainable and efficient continuous processes for oxidative biotransformations using Pickering emulsions (PE). By enabling the enzymatic production of peracids, the first step in lipase‐catalyzed oxidative functionalization, the process addresses key limitations of previously explored green reaction systems, such as enzyme deactivation and low specific activity. The PE system enhances emulsion stability and preserves enzyme activity under high oxidant concentrations, achieving high yields and space‐time productivity in a membrane‐based continuous reactor. Surface‐modified silica nanoparticles further improve interfacial catalytic efficiency as well as emulsion stability. This approach is well‐suited for selective and eco‐friendly oxidation in the synthesis of fine chemicals, active pharmaceutical ingredients, and specialty materials. Additionally, the robustness of the system allows stable operation under harsh conditions, supporting the efficient integration of the second, chemical epoxidation step. These findings contribute to the broader implementation of continuous green chemistry technologies in industrial biocatalysis.

**Abbreviations**
CalB: *Candida Antarctica* Lipase B
L/L: Liquid/liquid
MR: Membrane reactor
PE: Pickering emulsion
UHP: Urea hydrogen peroxide
w/o: Water‐in‐oil
wt: Weight

## 1 Introduction

The rising need for environmental conservation and sustainability has sparked significant interest in exploring alternative approaches to industrial oxidative functionalization, a crucial process for synthesizing various compounds, which often relies on toxic and hazardous oxidants [1, 2]. These alternative more benign routes involve the replacement of the toxic oxidants with green oxidants facilitated by enzyme catalyzed reactions [3]. For instance, investigations into the replacement of the traditional synthesis of epoxides (Prileschajew reaction), which are very important industrial chemicals [4], typically produced through oxidative functionalization processes involving hazardous and unstable peracids or strong mineral acids with lipase catalyzed routes have been gaining much attention [3, 4, 5, 6].

Lipase catalyzed oxidative functionalization of alkenes proceeds through a two‐step reaction, which involves the enzymatic oxidation of carboxylic acids with green oxidants to produce peracids in situ and the subsequent spontaneous oxidation of alkenes by the peracids [3, 7]. This in situ peracids synthesis efficiently catalyzed by *Candida antarctica* Lipase B (CalB) [6, 8, 9, 10], reduces the risk of handling the hazardous peracids as well as production cost [11]. However, the suitability of these environmentally friendly routes for industrial application is limited by challenges such as enzyme deactivation and/or low specific reaction rates [3, 5, 7]. The issue of enzyme deactivation observed in two‐phase [7, 12, 13] and solvent‐free reaction systems [14, 15] was significantly reduced when lipase‐catalyzed oxidative functionalization was investigated in anhydrous [12, 16, 17] and single‐phase systems [3, 5]. However, the anhydrous system showed low conversion efficiency [12], while the single organic phase system exhibited lower specific reaction rates compared to a single‐phase system containing an organic solvent like isopropanol, which is miscible with water and all required substrates [3, 5]. In this isopropanol‐based single‐phase system, however, the relative activity of the enzymes declined to less than 50% after six cycles [5].

For over a decade, Pickering emulsions (PEs), which are emulsions stabilized by nanoparticles, have gained significant attention as effective reaction media for multiphasic biocatalytic reactions due to their unique interfacial stabilization mechanism, making them particularly attractive for lipase‐catalyzed oxidative functionalization processes [18, 19, 20]. The adsorption of particles at the liquid–liquid (L/L) interface in PEs provides enhanced interfacial robustness, resulting in highly stable droplets that resist coalescence even under continuous mixing and oxidative conditions, thereby creating a protective microenvironment for enzymes against deactivation [9, 18, 21, 22, 23]. Additionally, the tunability of the particle surface chemistry enables strategic localization of enzymes at the interface, improving substrate accessibility and thereby enhancing catalytic efficiency, particularly in reactions that involve interfacial mass transfer [24, 25]. Recent studies by Hou et al. further demonstrated the potential of tailored nanoparticle design to improve PE stability [26]. They developed Janus cellulose nanocrystals via surface‐initiated atom transfer radical polymerization that exhibited enhanced interfacial anchoring and emulsion stability [26]. In addition, the expanding application of PEs in functional material development has been highlighted by their use as conductive and photothermal liquid‐metal inks [27] and as templates for photothermal phase‐change microcapsules [28], emphasizing the versatility of PE platforms across a wide range of engineering applications.

Given these advantages of PE, the synthesis of peracids which is the first, enzymatic step of lipase catalyzed oxidative functionalization of alkenes was investigated in PE in our previous work [9]. A specific reaction rate of 12.2 mmol h^−1^g^−1^ was achieved, which is over four times more than previously reported for a single‐organic phase system. Additionally, after 100 h of continuous peroxidation, the concentration of peroxyacetic acid was 92% that of the initial analyzed concentration when steady state was reached [9]. Despite this remarkable specific reaction rate and enzyme stability, the maximum yield in our previous studies was limited to 62%. Therefore, further investigations to optimize higher yields as well as reaction rates and enzyme stability are essential, particularly for the incorporation of the second step in lipase catalyzed oxidative functionalization.

Although PEs provide a protective interfacial environment that enhances enzyme stability [9], the stability of the emulsion decreases when the enzymes are present in the aqueous phase for water‐in oil (w/o) PE, leading to low catalytic activity [29]. Plikat et al. reported that this PE destabilization can be mitigated, albeit only slightly, by increasing the concentration of the nanoparticles [29]. However, this approach could impact the filterability of the system due to denser cake formation at the membrane surface, which results in increased energy cost [19, 29, 30]. A more effective strategy to address this destabilization of PE could be the immobilization of enzymes onto the nanoparticles. Hence, the nanoparticles function as both stabilizers and enzyme carriers, facilitating the direct placement of enzymes at the L/L interface. This dual role helps improve the stability of the emulsion and enhances the catalytic efficiency by maintaining enzyme activity at the interface where reactions occur while also protecting the enzyme [29, 31, 32]. In this work, the influence of differently modified HDKH2000 nanoparticles through silanization and coating with silicone‐based materials of different hydrophobicity on the enzymatic synthesis of peracids was investigated. The modification of the nanoparticles is essential for the effective immobilization of the enzyme onto the nanoparticles [31].

Building on the benefits of continuous processes [8], the efficient L/L separation by filtration of PE using a membrane reactor (MR) [19] and the findings from our previous study [9], this work evaluates other essential PE composition and operational parameters that influence the enzymatic step of lipase catalyzed oxidative functionalization of alkenes in w/o PE using a continuous MR integrated with an efficient flux control system. Additionally, this research will offer valuable insights for integrating the second step, which is the rate limiting step [14], and for modeling the entire process. Seiler et al. reported that the pH and salt concentration of the buffer (aqueous phase) in w/o PE significantly influences the catalytic activity of lipases [33]. Hence, this work particularly investigates how variations in PE composition such as pH and buffer concentration influence the synthesis of peroxyacetic acid. The slow and controlled release of hydrogen peroxide by urea hydrogen peroxide (UHP) makes it a suitable oxidant for enzymatic peroxidation especially for sensitive enzymes [16, 34]. Therefore, this study examined the use of UHP as an oxidant in comparison to aqueous hydrogen peroxide to assess its impact on reaction performance. As in our previous study, ethyl acetate was used as the continuous phase of the w/o PE in this work as it serves as both the excess medium and substrate for enzymatic peroxyacetic acid synthesis.

## 2 Materials and Methods

### 2.1 Materials

Organophilic nanofiltration membrane (oNF‐3, 900 Da) was purchased from BORSIG Membrane Technology GmbH, Germany.

Lyophilized lipase B from *Candida antarctica* (CalB lyo) with specific activity of 58,500 TBU/g was purchased from c‐Lecta GmbH, Germany. Ethyl acetate (≥99.8% for HPLC) and the chemicals used for the preparation of different concentrations and pH of phosphate buffer: di‐sodium hydrogen phosphate dihydrate (≥99.5%) and sodium dihydrogen phosphate dihydrate (≥99.8%) were purchased from Th. Geyer GmbH & Co. KG, Germany. Urea hydrogen peroxide adduct (97%) and the chemicals used for determining the concentrations of products and reactants through HPLC analysis as well as the derivatization: methyl p‐tolyl sulfide (MTS 97%), triphenylphosphine (TPP 99%), methyl p‐tolyl sulfoxide (MTSO 98%), and triphenylphosphine oxide (TPPO 99%) were purchased from Thermo Fischer Scientific GmbH, Germany. Hydrogen peroxide (50 wt% in water) was purchased from Sigma‐Aldrich GmbH, Germany. Silica nanoparticles HDKH2000 (25% SiOH) were donated by Wacker Chemie AG, Germany. Silica nanoparticles HDKH2000 were modified through salinization with tetra‐methyl oxazolidine and silicone‐based coating materials of different hydrophobicity as detailed in our recently published work [35], which also provides comprehensive morphological and physicochemical characterization confirming successful surface modification and changes in hydrophobicity essential for PEs stabilization.

### 2.2 Determination of the Partition Coefficient of the Oxidants

The partition coefficient of aqueous hydrogen peroxide between water and ethyl acetate was taken from Meyer et al. [6], reported as 0.1188. The partition coefficient of UHP between the organic and aqueous phase was determined by preparing solutions of known concentrations of urea hydrogen peroxide in deionized water. Ethyl acetate was added to each solution and the two phases were mixed using a rotor/stator homogenizer (IKA T25 digital UltraTurrax, Germany) with dispersing tool IKA S25N‐10G for 2 min at 17,600 min^−1^. The concentration of UHP in the organic phase was measured and the concentrations of UHP in the aqueous phase were calculated using Equation 2 below. Equation 2 was derived from the overall mass balance of UHP between the two phases (Equation 1).

(1) $$N_{UHP}^{total}=N_{UHP}^{org}+N_{UHP}^{aq}\;$$

(2) $$C_{UHP}^{aq}=\frac{N_{UHP}^{total}-V^{org}C_{UHP}^{org}}{V^{aq}}\;$$

The partition coefficient, *K*_UHP_ was determined from the plot of $C_{\mathrm{UHP}}^{\mathrm{org}}$ against $C_{\mathrm{UHP}}^{\mathrm{aq}}$ (Equation 3)

(3) $$C_{UHP}^{org}=K_{UHP}C_{UHP}^{aq}$$

### 2.3 Influent Solution Preparation

The influent solution was ethyl acetate saturated with the oxidant. For UHP, the UHP powder was dissolved in deionized water to the desired concentration. The solution was mixed with ethyl acetate (phase fraction of 0.5) for 2 mins, using the rotor/stator homogenizer with dispersing tool IKA S25N‐10G at 17,600 min^−1^ such that the ethyl acetate was saturated. The aqueous phase was discharged after the separation of the phases and the organic phase was used as the feed solution. For hydrogen peroxide, the influent solution was prepared in accordance to [6]. The partition coefficients of the oxidants, *K*_UHP_ and $K_{H_{2}O_{2}}$ were used to calculate the concentration of UHP and hydrogen peroxide respectively in their corresponding influent solution using Equation 4 (derived from Equation 2 and 3)

(4) $$C_{oxidant,in}^{org}=\frac{K_{oxidant}\frac{N_{H_{2}O_{2}}^{total}}{V_{aq}}}{1+K_{oxidant}\frac{V_{org}}{V_{aq}}}\;$$

### 2.4 Pickering Emulsions Preparation

3%wt/wt_dp_ of modified or unmodified HDKH2000 silica nanoparticles were dispersed in ethyl acetate and 1–10 gL^−1^_dp_ of CalB lyo was dissolved in phosphate buffer of different salt concentrations (10–300 mM) and different pH (5–9). The oxidant (hydrogen peroxide or UHP) was added to the ethyl acetate. The volume ($V_{H_{2}O_{2}}$) or mass (m_UHP_) of the oxidant added to the ethyl acetate was calculated such that the concentration of the oxidant in the influent solution equals the concentration of the oxidant in the continuous phase of the PE to be prepared at the start of the experiment [9] as shown in Equation 5 and 6 (derived from Equation 4). The two phases were homogenized using the rotor/stator homogenizer with dispersing tool IKA S25N‐10G for 2 min at 17600 min^−1^ which have been shown to be sufficient for the preparation of stable PEs [9]. An aqueous phase volume fraction (*φ*_dp_) of 0.3 was chosen for all experiments since in our previous work we found that compared to both higher and lower phase fractions the obtained PEs were more stable against coalescence when subjected to continuous stirring and pressure [9] and that the flux could be maintained at an industrially relevant level (> 20 Lm^−2^h^−1^).

(5) $$m_{UHP}=\frac{V_{aq}C_{UHP,in}^{org}{\tilde{M}}_{UHP}\left(1+\frac{K_{UHP}V_{org}}{V_{aq}}\right)}{K_{UHP}\cdot wt\%}$$

(6) $$V_{H_{2}O_{2}}=\frac{V_{aq}C_{H_{2}O_{2},in}^{org}{\tilde{M}}_{H_{2}O_{2}}\left(1+\frac{K_{H_{2}O_{2}}V_{org}}{V_{aq}}\right)}{\rho_{org}K_{H_{2}O_{2}}.wt\%}$$

### 2.5 Drop Size Determination

The droplet sizes of the prepared PEs before and after the reaction experiment were measured by taking 10 to 25 microscopic pictures of at least 500 droplets using the Axio Scope A1 Microscope (Carl Zeiss AG, Germany) and analyzing the images with an image analysis software (SOPAT GmbH, Germany). The Sauter mean diameter (d_3,2_) was calculated from the droplet diameter (*d*_drop,i_) using Equation (7).

(7) $$d_{3,2}=\sum_{i=1}^{n}d_{drop,i}^{3}/\sum_{i=1}^{n}d_{drop,i}^{2}\;$$

### 2.6 Set‐up of Membrane Reactor

**FIGURE 1:**
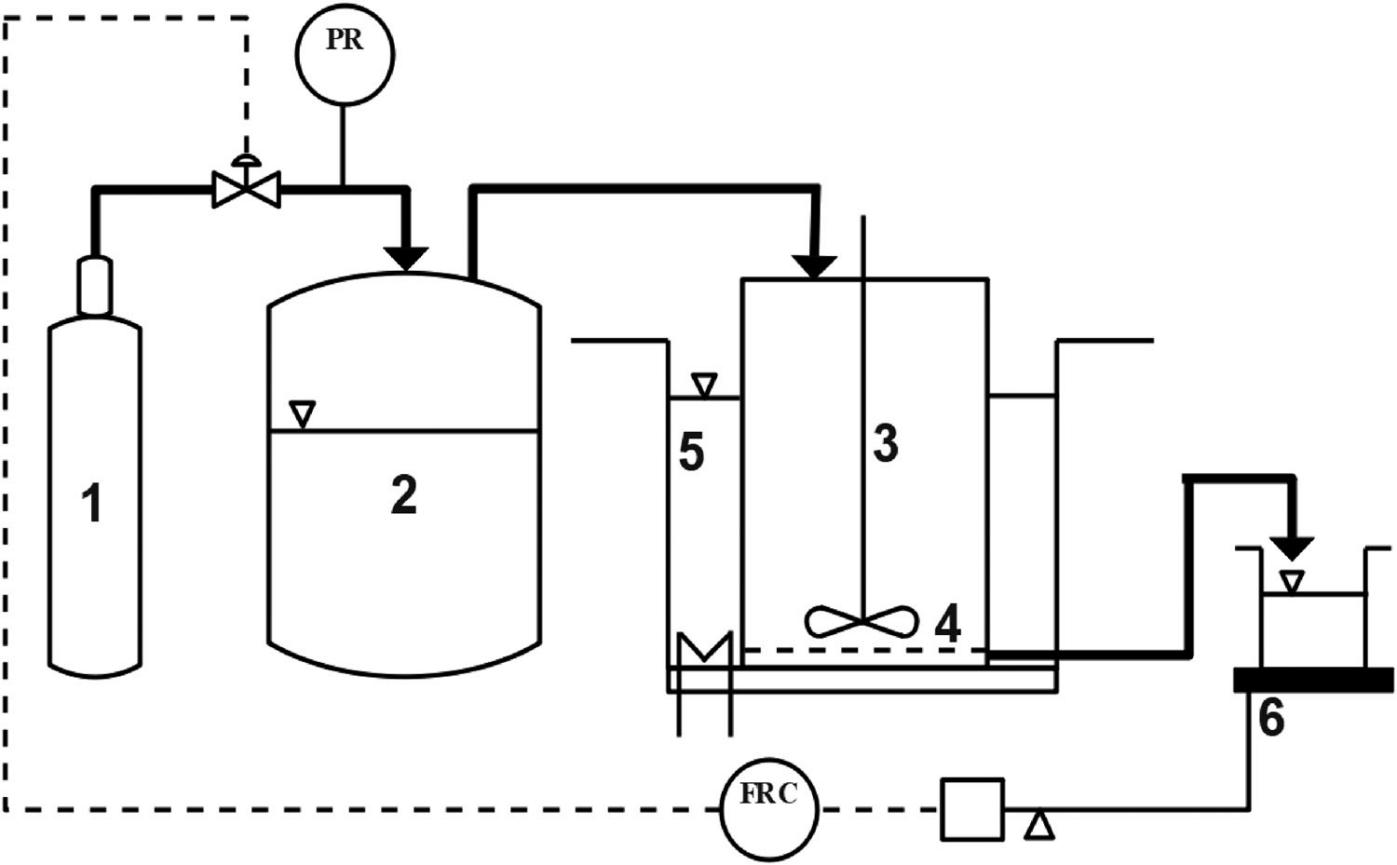
Flowsheet of the continuously stirred membrane reactor system. Nitrogen gas tank (1), feed tank (2), reactor (3), membrane (4), water bath (5), and electronic balance (6).

The reactor set up is shown in Figure 1. A continuously stirred membrane reactor of volume, *V*_MR_ = 87 mL and effective area, *A*_eff_ = 13.2 cm^2^ suited for 47 mm membrane disc was used for the continuous synthesis [9]. An organophilic nanofiltration membrane (oNF‐3, 900 Da) purchased from BORSIG Membrane Technology GmbH, Germany, was used [9, 36].

The collected effluent was weighed using a VWR LPW‐723i electronic precision balance (VWR, GmbH, Germany). The Laboratory Virtual Instrument Engineering Workbench (LabVIEW) professional software, version 12.0 (32‐bits), 2012 was used to control the continuous process and saved the collected data (effluent weight, flux, volume flow, pressure, and time). A Proportional‐Integral‐Derivative (PID) controller was set up in the LabView software to control the process flux. The tuning of the PID parameters was done in accordance to [37, 38]. The resulting tuned PID values were *K*_p_ = 4 bar.m^2^hL^−1^, *T*_i_ = 36.7 s and *T*_d_ = 8.9 s. Additionally, for various set flux values (10 – 30 Lm^−2^h^−1^), the steady‐state error was consistently below 5%, with steady state being reached within 4 min (Supporting information, Table S1 and Figure S2).

### 2.7 Continuous Peroxyacetic Acid Synthesis

At the start of each experiment, the reactor was filled with the prepared PE, after which the stirrer speed was set to 500 min^−1^ and temperature was set to between 24^°^C and40^°^C. The continuous peroxyacetic acid synthesis was carried out at constant flux (hence *τ* = constant, Equation 8). For this, the required flux is entered into the LabVIEW program which sends a signal to open the valve such that the appropriate pressure is released from the gas bottle. The influent solution from the feed tank is then fed into the reactor such that the same amount of effluent is collected by the product tank on the electronic balance. The flux (J) was calculated by the LabVIEW program from the change in the effluent mass collected by the electronic balance every 5 s using Equation 9. The difference between the calculated and set flux builds an error which sends a signal to the valve to either increase or decrease its opening for the appropriate pressure to be released. With this continuous cycle, the flux was controlled throughout the experiment.

(8) $$\tau =\frac{V_{MR}}{\dot{V}}$$

(9) $$J=\frac{\left(m_{i+1}-m_{i}\right)}{A_{eff}\;\rho_{effluent}\left(t_{i+1}-t_{i}\right)}$$

Once steady state was reached, determined in our previous work as 1*τ* [9], effluent analysis was carried out at 20‐min intervals until the peroxyacetic acid concentration shows a standard deviation *σ*<7.5% for at least three consecutive samples. Two reactors were set up to give duplicate result for each experiment. The rate of reaction, *Ṙ*_steady_, specific rate of reaction *ṙ*_steady_, yield, *Y*_steady_, conversion, *X*_steady_, space‐time yield, *STY*_steady_ and catalytic productivity, *P*_enz_ at steady state were determined using Equations ((10), (11), (12), (13), (14), (15)) respectively.

(10) $${\dot{R}}_{steady}=\frac{C_{PA,out}}{\tau }$$

(11) $${\dot{r}}_{steady}=\frac{V_{MR}.{\dot{R}}_{steady}}{m_{CalB}}\;$$

(12) $$Y_{steady}=\frac{C_{PA,out}}{C_{H_{2}O_{2},in}}\;$$

(13) $$X_{steady}=\frac{C_{H_{2}O_{2},in}-C_{H_{2}O_{2},out}}{C_{H_{2}O_{2},in}}\;$$

(14) $$STY_{steady}={\dot{R}}_{steady}\cdot \tilde{M}$$

(15) $$P_{CalB}=\frac{m_{PA,out}}{m_{CalB}}$$

Given that the enzyme‐reactants interactions in PE occur predominantly at the L/L interface [39], the area‐specific reaction rate $\left({\dot{r}}_{A}\right)$ was used to evaluate the effect of PE composition on enzyme performance. Since the interfacial area (*A*_int_) is influenced by droplet size, which varies with PE composition [9], it was estimated using the aqueous phase volume and the Sauter mean diameter (Equation 16). The corresponding area‐specific reaction rate was then calculated using Equation 17. This approach delineates the influence of the investigated PE composition on the enzyme's catalytic activity and behavior at the interface [33].

(16) $$A_{int}=\frac{6V_{aq}}{d_{3,2}}$$

(17) $${\dot{r}}_{A}=\frac{{\dot{R}}_{steady}}{A_{int}}$$

To account for changes in droplet size during the reaction, the area‐specific reaction rate was calculated using the interfacial area estimates obtained at the start of the experiment and at the point when pseudo‐steady state was reached (1τ), as the droplet size was only measured at these two time points. The actual value is assumed to lie between these bounds, reflecting the dynamic nature of the emulsion an enabling a more representative assessment of enzyme performance at the interface.

### 2.8 Effluent Analysis

For the simultaneous determination of the concentration of hydrogen peroxide or UHP and peroxyacetic acid in the effluent, a derivatization reaction, as carried out in our previous work [9], in accordance to [6] was used. A varian ProStar HPLC system (Varian, Inc., USA) fitted with a Varian ProStar 335 photodiode array detector, Varian 460‐LC autosampler and OTU Trikala C18,105Å, 5μ, 150 × 3.0 mm column (Applichrom GmbH, Germany) was used for the analysis. An isocratic system consisting of 65% acetonitrile and 35% water by volume was used at a constant flowrate of 1 mL/min. UV detection was performed at a wavelength of 230 nm, with an injection volume of 20 µL. The Varian MS workstation (version 6.9.3) was used for system control and data acquisition. Calibration curves exhibited linearity (*R* ^2^> 0.99) within the ranges of 0.004 to 0.47 mM for MTSO and 0.01 to 0.16 mM for TPPO (Figure S1) at retention times of 0.95 and 1.45 min, respectively.

## 3 Results and Discussion

### 3.1 Partition Coefficient of UHP

To ensure that the concentration of UHP in the organic phase (ethyl acetate) of the prepared PE at the start of the reaction equals the concentration in the feed tank, and considering that the feed to the reactor is the organic phase saturated with the oxidant, we determined the partition coefficient of UHP between the organic and aqueous phase.

**FIGURE 2:**
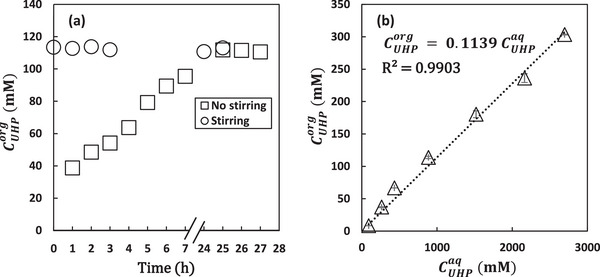
a) Concentration of urea hydrogen peroxide (UHP) in ethyl acetate over time with and without stirring of the organic and aqueous phase (*N* ^total^ = 0.002 mols) b). Partition coefficient of UHP between ethyl acetate and water. Experimental conditions: *V*_org_ = 2 mL, *V*_aq_ = 2 mL, *n* = 17,600 min^−1^, *T* = 20°C. Error bars represent the standard deviation from the mean values from duplicate experiments.

Figure 2a shows that when the two phases were added together without stirring, it takes more than 7 h for the organic phase to be saturated with UHP. However, when the two phases were mixed at 17,600 min^−1^ for 2 mins, saturation was achieved instantaneously (Figure 2a). To determine the partition coefficient, the two phases were mixed at 17,600 min^−11^ for at least 2 mins for different concentrations of UHP. To avoid errors caused by excessive dilution of samples [6], the concentration of UHP in the aqueous phase was determined using Equation 2 rather than being analyzed by HPLC.

Figure 2b shows the partition coefficient of UHP (*K*_UHP_ = slope) between the two phases as 0.1139. This was determined over a linear concentration range of UHP in ethyl acetate from 8.4–303 mM, with a coefficient of determination, *R* ^2^> 0.99. This slightly lower partition coefficient, compared to that of aqueous hydrogen peroxide (0.1188) [6], may be due to the higher polarity of UHP. The presence of urea in UHP introduces additional polar groups, increasing UHP's affinity for the aqueous phase, making it less likely to partition into the organic phase.

### 3.2 Influence of PE Composition

In our previous work it was shown that the composition of the PE, such as enzyme concentration, L/L phase fraction, and nanoparticle type and concentration, significantly affects peroxyacetic acid synthesis, although droplet size had minimal direct impact [9]. In this study, other aspects of PE composition such as buffer pH and salt concentration [33], which influence both peroxyacetic acid synthesis and droplet size were examined. In addition, the influence of differently modified nanoparticles, as stabilizers/carriers to facilitate direct interfacial placement of the enzymes, on emulsion stability and reaction performance was assessed to identify the most effective modification strategy. Unlike earlier experiments conducted under constant pressure [9], enzyme concentration was evaluated here under constant flux to maintain a stable hydraulic retention time.

#### 3.2.1 Influence of pH and Salt Concentration of the Buffer

**FIGURE 3:**
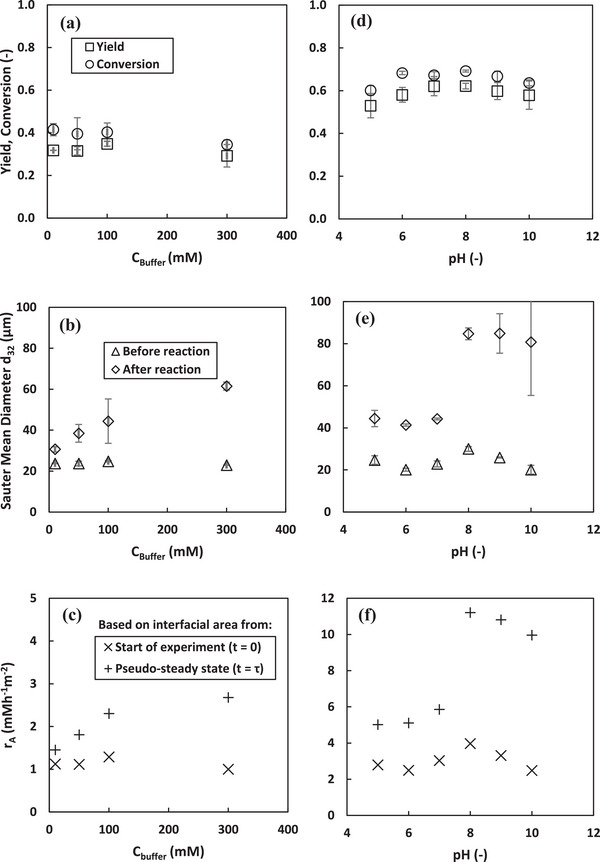
Influence of aqueous phase composition on peroxyacetic acids synthesis, Sauter mean diameter and area specific reaction rate at pseudo‐steady state in a continuous peroxidation process. (a–c): Effect of buffer concentration. Experimental conditions: *T* = 30°C, τ = 4.6 h, $C_{H_{2}O_{2},\;\mathrm{in}}$ = 106 mM, *C*_CalB_ = 1 gL^−1^_dp_. (d–f): Effect of pH. Experimental conditions: *T* = 24 ^0^C, *τ* = 4.6 h, $C_{H_{2}O_{2},\;\mathrm{in}}$ = 153 mM, *C*_CalB_ = 5 gL^−1^_dp_. Error bars represent the standard deviation from the mean values from duplicate experiments.

Enzymes are sensitive to variations in pH which can directly influence their activity [39]. Hence, buffers are essential in the aqueous phase of bioactive w/o PE to maintain the pH, particularly since the enzymes adsorb at the L/L interface while some might remain in the aqueous phase. However, the salt concentration of the buffer impacts not only the enzyme activity but also the stability of the emulsion against coalescence [33, 40]. Figure 3b shows that with increasing salt concentration of the buffer, the bioactive PE were less stable against coalescence when subjected to continuous stirring and pressure. This is consistent with the results obtained by [40, 41, 42], which was attributed to the increased electrostatic screening of the interactions between the particles and the interface with increasing salt concentration of the buffer, which facilitates coalescence during continuous stirring and pressurization. Additionally, the increased concentration of the salts can reduce the trapping energy of the particles at the interface [40]. However, Ribeiro et al. reported that even 7 days after preparation, PE prepared using a more hydrophobic sunflower oil as the organic phase and stabilized by nano‐hydroxyapatite remained stable against coalescence despite increasing the salt concentration from 0 to 500 mM [43].

Figure 3e shows that the stability of the emulsions against coalescence is influenced by the pH of the aqueous phase under continuous stirring and pressure. Between pH 5 and 7, no significant difference in the stability of the prepared PE against coalescence was observed. However, as the aqueous phase becomes more alkaline (pH 8–10), the PE becomes less stable against coalescence. This is consistent with results reported by Cai et al., in which PE prepared under alkaline conditions was less stable than those prepared under neutral or acidic conditions, even 9 days after preparation [44]. They attributed this reduced stability to a decrease in electrostatic repulsion between droplets under alkaline conditions, which promotes droplet flocculation and subsequently leads to coalescence. Additionally, the pH‐dependent stability of the bioactive PE may be influenced by electrostatic interactions between CalB and the nanoparticles, as well as potential changes in the stabilizing particles wettability under alkaline conditions [33]. The strong deprotonation of the particles surface under alkaline conditions may reduce surface hydrophilicity, leading to less stable emulsions.

The influence of the pH and salt concentration of the buffer on the enzyme activity was evaluated by determining the catalytic productivity (*P*_CalB_) at various pH and buffer concentrations (at pH 7). Table 1 shows that increasing the buffer concentration from 10 to 100 mM resulted in a slight increase in the catalytic productivity, from 1.33–1.46 g_PA_g^−1^_CalB_h^−1^. However, further increase in the concentration to 300 mM led to a 16% decrease in the catalytic productivity (1.23 g_PA_g^−1^_CalB_h^−1^). Additionally, Figure 3a shows that the product yield was highest at a buffer concentration of 100 mM. Hence at pH 7, a buffer concentration of 100 mM provides optimal pH stability for the enzymatic synthesis of peroxyacetic acids in PE. At lower concentrations (< 100 mM), the pH likely drops below 7 due to the formation of acetic acid as an intermediate product [9], which may alter the local pH in the aqueous phase and near the L/L interface. On the other hand, higher buffer concentrations (>100 mM) could lead to dehydration of the CalB, a protein, due to the hygroscopic nature of phosphate salts [45, 46]. This dehydration, combined with the high salt strength, can disrupt electrostatic interactions at the enzyme's active site, reducing its catalytic efficiency.

**TABLE 1:** Composition of PE and operational parameters influence on the synthesis of peroxyacetic acid. *C*_particles_ = 3%wt/wt_dp_, *φ* = 0.3.

	PE composition				Operational parameters			Results			
	C_buffer_	pH	Nanoparticles	*C*_CalB_	*T*	*τ*	*C*_oxidant, in_	*Ṙ*_steady_	*ṙ*_steady_	*STY*_steady_	*P*_CalB_
	[mM]	[‐]	[‐]	[gL^−1^_dp_]	[^0^C]	[h]	[mM]	[mMh^−1^]	[mmolh^−1^g^−1^]	[g_PA_L^−1^d^−1^]	[g_PA_g^−1^_CalB_h^−1^]
Figure 3a	10 50 100 300	7	Native	1	30	4.6	106	7.4	25.1	13.5	1.33
								7.3	24.9	13.4	1.33
								8.1	27.5	14.8	1.46
								6.8	23.1	12.4	1.23
Figure 3d	100	5	Native	5	24	4.6	153	17.7	12.1	32.2	0.64
		6						19.4	13.3	35.3	0.71
		7						20.7	14.2	37.8	0.76
		8						20.7	14.2	37.8	0.76
		9						19.9	13.7	36.4	0.73
		10						19.3	13.2	35.2	0.70
Figure 4a	50	7	Native	1	30	4.6	289	22.0	77.3	40.2	4.12
			Silanized					19.9	69.8	36.3	3.72
			SIL‐VS‐PEG					20.8	72.9	37.9	3.88
			SIL‐PEG					20.1	70.4	36.6	3.75
			SIL‐VS					20.1	70.7	36.8	3.76
Figure 6a	100	7	Native	1	24	6.9	106	6.7	22.9	12.3	1.22
				3				10.4	11.7	18.9	0.62
				5				11.1	7.5	20.2	0.40
				10				11.1	3.8	20.2	0.20
Figure 7a	100	7	Native	1	24	4.6	106	8.1	27.4	14.8	1.46
					30			8.1	27.5	14.8	1.46
					40			4.4	14.9	8.0	0.79
Figure 7c	100	7	Native	5	24	14.7	106	5.8	3.9	10.6	0.21
						10.1		8.1	5.5	14.9	0.29
						6.9		11.1	7.5	20.2	0.40
						4.7		14.5	9.8	26.5	0.52
						3.8		17.4	11.8	31.7	0.63
						3.4		18.7	12.7	34.1	0.67
Figure 7e	100	7	Native	5	24	6.9	45	5.4	3.6	9.9	0.19
							106	11.1	7.5	20.2	0.40
							196	18.6	12.9	34.0	0.68
							386	24.6	17.5	44.9	0.93
							758	21.4	16.3	39.1	0.87
							1685	12.4	10.8	22.6	0.57
Figure 7e	100	7	Native	5	24	6.9	196	18.5	12.4	33.7	0.66
(UHP)							386	21.0	14.2	38.3	0.76

Table 1 shows that the catalytic productivity of CalB was optimal at pH 7 and 8 (0.76 g_PA_g^−1^_CalB_h^−1^), indicating that Lipase B performs best in a neutral to slightly alkaline environment. However, as the pH decreased to 5 (acidic conditions), the catalytic productivity dropped by 15%. Similarly, in a more alkaline environment (pH 10), the catalytic productivity declined to 0.70 g_PA_g^−1^_CalB_h^−1^. Although this study focused on CalB‐catalyzed oxidative functionalization, these trends are in line with previous findings on CalB activity in hydrolysis and transesterification reactions, which reported optimal activity between pH 7 and 7.5 [47, 48, 49]. Seiler et al. also observed that the pH of lipases' highest activity varied depending on buffer concentration [33]. In this study, peak activity at pH 7 and 8 was observed at a buffer concentration of 100 mM. Although the isoelectric point of Lipase B is at pH 6, the peak activity in this study near this value may result from CalB having no net charge between pH 5 and 8 [39]. This is consistent with Seiler et al. who reported that lipases exhibit highest activity at or close to their isoelectric points across different buffer concentrations [33]. The results (Figure 3d and Table 1) further indicate that CalB is sensitive to structural and electrostatic changes induced by pH variations in the environment.

Figure 3c shows that although smaller droplet sizes, hence larger interfacial areas were observed at lower buffer concentrations after the reaction (Figure 3b), the average area‐specific reaction rate was highest at 100 mM buffer concentration. This aligns with the trend observed in catalytic productivity at varying buffer concentrations and suggests that enzyme performance rather than interfacial area, was the dominant factor influencing reaction efficiency. Although catalytic productivity at pH 7 and 8 was the same, Figure 3f shows that the optimal area‐specific reaction rate was obtained at pH 8. This was 31% higher than at pH 7 when calculated using the initial interfacial area, and increased to 92% when based on the larger Sauter mean diameter difference observed at pseudo‐steady state (Figure 3e). However, in a more alkaline environment at pH 10, the area‐specific reaction rate declined relative to pH 8 by 37.5% when calculated using the initial interfacial area, and by 11.1% when based on droplet size at pseudo‐steady state. This reduction may be due to changes in enzyme stability and interfacial properties under alkaline conditions, leading to less effective catalysis despite comparable droplet sizes at pseudo‐steady states. Similarly, as shown in Table 1 for the catalytic productivity, a decline in area‐specific reaction rate was also observed under more acidic conditions compared to pH 7, despite comparable droplet sizes between pH 5 and 7 (Fig 3e). These observations indicate that the reaction rate changes were primarily due to pH‐induced effects on enzyme activity rather than differences in interfacial area. However, taking into consideration the stability of the emulsion against coalescence and catalytic productivity, subsequent investigations were done at pH 7 with buffer concentration of 100 mM.

#### 3.2.2 Influence of Modified Nanoparticles

**FIGURE 4:**
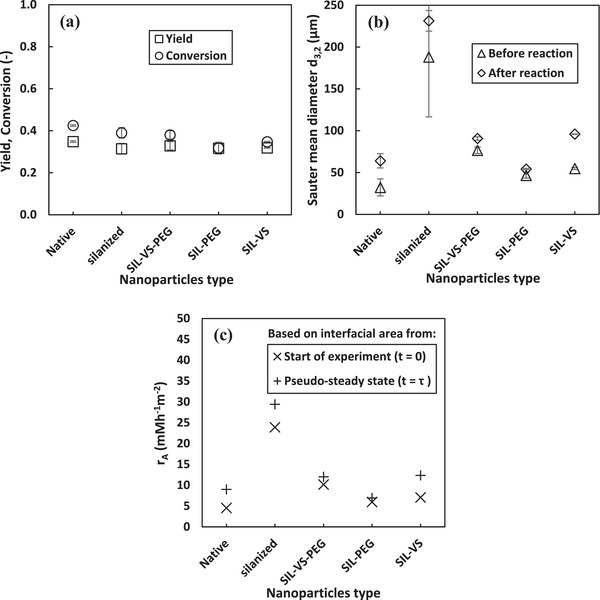
Influence of modified silica nanoparticles on peroxyacetic acids synthesis (a), Sauter mean diameter (b) and area specific reaction rate (c) at pseudo‐steady state in a continuous peroxidation process. Experimental conditions: *T* = 30 ^0^C, *τ* = 4.6 h, $C_{H_{2}O_{2},\;\mathrm{in}}$ = 289 mM, *C*_CalB_ = 1 gL^−1^_dp_. Error bars represent the standard deviation from the mean values from duplicate experiments.

Figure 4 shows the influence of modified HDK H2000 nanoparticles on the stability PE and peroxyacetic acid synthesis. Figure 4b shows that the Sauter mean diameter of PE stabilized by silanized nanoparticles was the largest, measuring 5.8 times greater than that of PE stabilized by unmodified nanoparticles (Native). This increase can be attributed to the significant reduction in particle wettability caused by silanization, which renders the surface more hydrophobic [50]. In general, PE stabilized with unmodified nanoparticles exhibited the smallest Sauter mean diameter. Furthermore, at the same mass, silanized and coated nanoparticles provide fewer particles for stabilization compared to unmodified ones, resulting in lower surface coverage [35]. However, PE stabilized with silanized nanoparticles exhibited greater resistance to coalescence (23% increase in droplet size) compared to those stabilized with unmodified nanoparticles (98.8% increase) when subjected to continuous pressure and stirring. This improved stability may be due to the silanization process reducing the surface energy of the particles, which enables the formation of irreversible adsorption layers at the interface, creating a more robust protective barrier against coalescence, even with fewer particles at the same mass [51].

**FIGURE 5:**
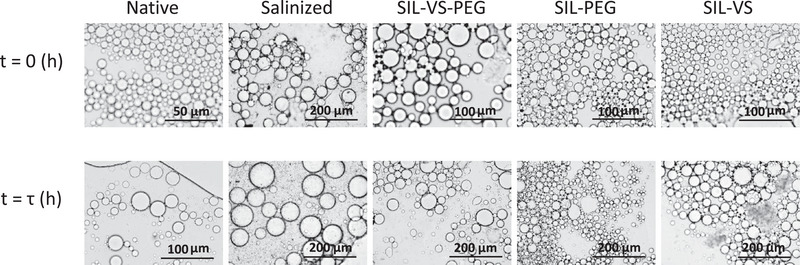
Optical microscopic images of w/o PEs stabilized by native and surface‐modified HDK H2000 nanoparticles before the reaction (*t* = 0 h) and at pseudo‐steady state (*t* = *τ*). Experimental conditions: *T* = 30 ^0^C, *τ* = 4.6 h, $C_{H_{2}O_{2},\;\mathrm{in}}$ = 289 mM, *C*_CalB_ = 1 gL^−1^_dp_, *φ* = 0.3, *C*_particles_ = 3%wt/wt_dp_.

Among the coated nanoparticles, the Sauter mean diameter of PE stabilized by particles coated with amphiphilic materials (SIL‐VS‐PEG) were the largest. In contrast, there was no significant difference in the droplet size of PE stabilized by nanoparticles coated with hydrophobic (SIL‐VS) or hydrophilic (SIL‐PEG) materials. However, the stability of PE against coalescence decreased as the hydrophobicity of the coating materials increased. This is likely because a more hydrophobic coating increases the hydrophobicity of the nanoparticles. This is consistent with the results reported by Kempin et al., in which PE stabilized with more hydrophobic nanoparticles were less stable against coalescence when subjected to continuous stirring and pressure [52]. The improved stability of PE stabilized by SIL‐PEG‐coated nanoparticles (16% increase in drop size) may be due to the hydrophilic coating providing better water affinity while maintaining the intermediate wettability required for w/o emulsions. Heidari et al. observed similar resistance to coalescence for nanoparticles optimally modified with hydrophilic chitosan, with drop size increasing by less than 15% over a 30‐day period [53]. Ultimately, PE stabilized by all modified nanoparticles were more stable against coalescence compared to unmodified nanoparticles. Figure 5 shows representative microscopic images of PEs stabilized by unmodified and modified nanoparticles at the start of the reaction and at pseudo‐steady state, demonstrating the improved stability against coalescence achieved with surface‐modified nanoparticles.

Figure 4a and Table 1 shows that there is no significant difference in product yield, reaction rate, or catalytic productivity for all reactions in PE stabilized by modified nanoparticles. However, PE stabilized with silanized nanoparticles exhibited the lowest reaction rate and catalytic productivity compared to those stabilized with unmodified and coated nanoparticles. This could be attributed to the significantly larger droplet sizes observed in PE stabilized by silanized nanoparticles. Consequently, a slightly higher reaction rate and catalytic productivity was observed in PE stabilized by unmodified nanoparticles due to their smaller drop sizes compared to those stabilized by modified nanoparticles. This minimal influence of drop size on the reaction is consistent with findings from our previous work, which showed that smaller drop sizes slightly enhance mass transfer due to increased surface area‐to‐volume ratio, thereby improving substrate‐enzyme interactions [9, 54].

Figure 4c shows that, with the exception of PE stabilized by SIL‐VS‐coated nanoparticles, the area‐specific reaction rate in all PE systems stabilized by other modified nanoparticles was higher than that observed for PE stabilized by native nanoparticles. Notably, reactions in PE stabilized by silanized nanoparticles exhibited the highest area‐specific reaction rate, which was 5.3 times greater than that of PE stabilized by native nanoparticles when calculated using the initial droplet size, and 3.3 times greater when based on the droplet size measured at pseudo‐steady state. This indicates that surface modification of nanoparticles enhances reaction efficiency at the interface, likely due to improved interfacial stability, stronger surface interactions, and/or better enzyme orientation for catalysis. Alternatively, the lower performance observed in PE stabilized by native nanoparticles may result from insufficient enzyme coverage at the interface. Given the relatively low enzyme concentration used in this experiment (gL^−1^_dp_) and the smaller droplet sizes in PE stabilized by native nanoparticles, corresponding to a larger interfacial area, it is possible that the interface was not fully saturated with enzyme. In contrast, the larger droplet sizes observed in PE stabilized by modified nanoparticles (i.e., smaller interfacial area) may have allowed for more complete enzyme coverage, contributing to higher area‐specific reaction rates. Among the coated nanoparticles, reactions in PE stabilized by SIL‐VS‐PEG‐coated nanoparticles exhibited the highest average area‐specific reaction rate, while reactions in PE stabilized by SIL‐PEG‐coated nanoparticles resulted in the lowest. This shows that SIL‐VS‐PEG‐coated nanoparticles provide an optimal balance between interfacial stabilization and enzyme accessibility compared to SIL‐VS‐ and SIL‐PEG‐coated nanoparticles. Overall, these results highlight the crucial role of nanoparticle surface modification in optimizing interfacial catalysis when used as stabilizers in PE. Additionally, modified nanoparticles show greater effectiveness for applications requiring high interfacial catalytic activity in PE.

#### 3.2.3 Enzyme Concentration

**FIGURE 6:**
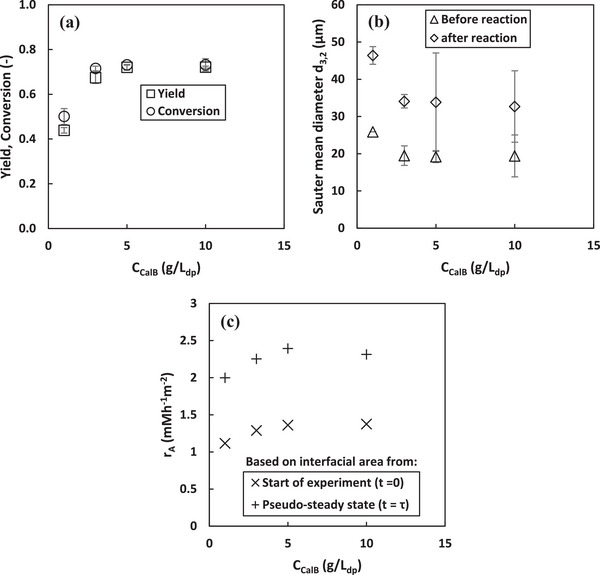
Influence of enzyme concentration on peroxyacetic acids synthesis, Sauter mean diameter and area specific reaction rate at pseudo‐steady state in a continuous peroxidation process. Experimental conditions: *T* = 24 ^0^C, *τ* = 6.9 h, $C_{H_{2}O_{2},\;\mathrm{in}}$ = 106 mM. Error bars represent the standard deviation from the mean values from duplicate experiments.

Figure 6b shows that increasing the enzyme concentration from 1 to 3 gL^−1^_dp_ led to a 24.8% reduction in the Sauter mean diameter while also enhancing resistance to coalescence. This trend is consistent with literature reports for CalB and *Candida antarctica* lipase A (CalA) [9, 55]. The observed reduction in droplet size and improved coalescence resistance were attributed to the reduction in interfacial tension with increasing lipase concentration [55]. However, as shown in Figure 6a, further increasing the enzyme concentration beyond 3 gL^−1^_dp_ had no significant impact on droplet size or coalescence resistance, likely due to interfacial saturation at this concentration.

Figure 6a shows that increasing enzyme concentration from 1 to 10 gL^−1^_dp_ at a substrate concentration of 106 mM resulted in a corresponding increase in product yield from 44% to 72%. However, no further increase in product yield or space‐time yield was observed beyond 5 gL^−1^_dp_. Additionally, Table 1 shows that increasing the enzyme concentration from 5 to 10 gL^−1^_dp_ reduced the catalytic productivity by 50%. This decline can be attributed to the increase in the rate of both the forward reaction and the undesired backward reaction with increasing enzyme concentrations, as shown in our previous study [9]. The undesired backward reaction produces acetic acid, which likely lowers the pH at the L/L interface and can also damage the enzyme structure [6].

Figure 6c shows an increase in the area‐specific reaction rate with increasing enzyme concentration. This trend differs from that of specific reaction rate and catalytic productivity (Table 1), suggesting that at lower enzyme concentrations (e.g., 1 gL^−1^_dp_), the enzyme likely does not fully saturate and hence utilize the available interfacial area for catalysis at this concentration, resulting in lower catalytic efficiency per unit area. Therefore, as the enzyme concentration increases, more enzyme molecules occupy the interface, enhancing catalytic efficiency per unit area. However, beyond 5 gL^−1^_dp_, further increasing the enzyme concentration does not significantly improve interfacial activity, indicating that saturation occurs at this concentration. This saturation effect can be attributed to constraints in enzyme distribution at the interface and/or substrate availability. To maximize product yield and interfacial activity while maintaining high catalytic efficiency and specific reaction rates, optimizing enzyme concentration is essential. Therefore, at a substrate concentration of 106 mM, increasing the enzyme concentration above 5gL^−1^_dp_ is uneconomical and can significantly reduce the overall system efficiency.

### 3.3 Influence of Operational Parameters

**FIGURE 7:**
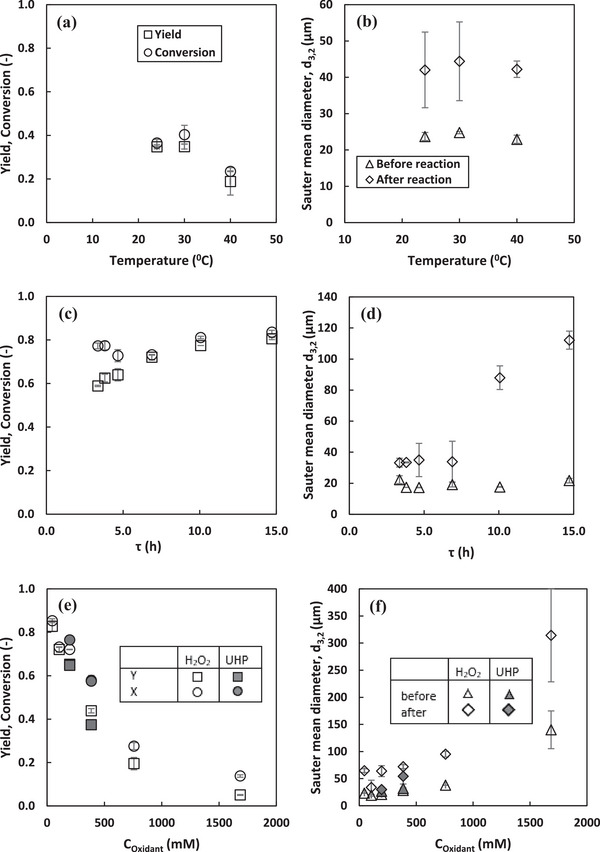
Influence of operating parameters on peroxyacetic acids synthesis and PE stability against coalescence at pseudo‐steady state in a continuous peroxidation process. Operating conditions are shown in Table 1. Error bars represent the standard deviation from the mean values from duplicate experiments.

For the efficient development of continuous lipase catalyzed oxidative functionalization of alkenes in PE, it is crucial to not only optimize the composition of PE but also identify the operational parameters and the extent to which these parameters impact the first, enzymatic step (peroxyacetic acid synthesis) as well as the stability of PE against coalescence. This information is essential for effectively integrating the second step of the reaction and for optimizing and modeling the entire process. Table 1 and Figure 7 show the effects of various operational parameters on the reaction performance as well as emulsion stability at pseudo‐steady state, which is reached after 1*τ*, as reported in our previous work [9].

#### 3.3.1 Temperature

Although enzyme activity generally increases with temperature, there is a limit beyond which enzyme stability decreases due to denaturation [56]. In the investigation of lipase‐catalyzed peroxyacetic acid synthesis in a single organic phase, complete enzyme deactivation was observed at 50°C even before pseudo‐steady state was reached [6]. Therefore, this study focused on a temperature range of 24°C–40°C. Figure 7b shows that increasing the reaction temperature from 24°C to 40°C does not influence the stability of the emulsions against coalescence. This is consistent with the findings of Ribeiro et al. and Mwangi et al., in which it was reported that up to 50°C, PE stability against coalescence remained unchanged, even after 7 days of storage [43, 57]. However, at higher temperatures, resistance to coalescence significantly decreased, likely due to the increased kinetic energy of the droplets, which promotes collisions and coalescence. In this study however, reactions at temperatures beyond 40°C were not investigated.

Figure 7a and Table 1 show that between 24°C and 30°C, the yield, specific rate of reaction, and catalytic productivity remained constant. However, as the temperature increased from 30°C to 40°C, there was a significant reduction in yield and catalytic productivity, dropping by 46%. This indicates a significant loss in enzyme activity, likely due to denaturation. Furthermore, at 40°C, the backward reaction likely becomes more prominent, with the decomposition of peroxyacetic acid surpassing its formation, resulting in a significantly lower yield. This finding is in contrasts with the results reported by Meyer et al., in which in a single organic phase system, the product yield for peroxyacetic acid synthesis was the same at both 30°C and 40°C but decreased marginally (by 8%) at 21°C [6]. This contrast highlights the different temperature sensitivities of enzymatic peroxyacetic acid synthesis in various reaction systems. In addition, the absence of an L/L interface in a single organic‐phase system might remove structural temperature limitations, allowing the enzymes to maintain activity over a broader range of temperatures. Moreover, the immobilized lipase used by Meyer et al. is less prone to temperature limitations compared to lyophilized enzymes used in this work [6]. Considering the yield, catalytic productivity, and overall process economics, a temperature of 24°C is recommended and was used for further investigations.

#### 3.3.2 Residence Time

Figure 7d shows the influence of residence time on the stability of the emulsion against coalescence, considering that droplet size analysis was conducted both at the start of the experiment (*t* = 0) and when pseudo‐steady state peroxidation was reached (*t* = *τ*). For residence times of 6.9 h and below, there was no impact on emulsion stability against coalescence as shown in Figure 7d. This suggests that within this timeframe, the nanoparticles effectively maintained interfacial coverage and stabilization. However, for residence times exceeding 6.9 h, the emulsion became significantly less stable against coalescence, with a 417% increase in droplet size at a residence time of 14.7 h, which is 3.3 times larger than at 6.9 h. This significant decrease in emulsion stability can be attributed to potential destabilization mechanisms such as partial desorption or restructuring of nanoparticles at the interface, compromising their stabilizing effect and thereby promoting coalescence. Additionally, longer residence times likely increase droplet interactions, which also increases the likelihood of coalescence.

Figure 7c and Table 1 show that hydraulic residence time significantly influences the reaction performance. As the residence time increases from 3.4 to 14.7 h, the yield increases substantially from 59%–81%. This outcome is expected as longer residence times allow for more extensive interaction between the enzyme and substrate at the L/L interface. However, shorter residence times enhance reaction rates, catalytic productivity, and space‐time yields, consistent with findings reported in literature [6] and as described by Equations (10), (14), and 15. Moreover, even at prolonged residence times of up to 14.7 h, no negative effects were observed from possible side reactions, enzyme deactivation or peroxyacetic acid decomposition. Maximum reaction selectivity (Table S2) was achieved at a residence time of 6.9 h, indicating an optimal balance between forward and backward reactions as well as possible side reactions. Furthermore, when considering strategies to mitigate enzyme deactivation, longer residence times, such as 6.9 h at low substrate concentrations not detrimental to the enzymes are advantageous. This condition was therefore adopted for subsequent investigations.

#### 3.3.3 Oxidant Type and Concentration

UHP has been reported as a highly suitable oxidant for epoxidation reactions due to its controlled release of hydrogen peroxide, which likely enhances emulsion and enzyme stability [16]. Figure 7f shows that increasing the concentration of aqueous hydrogen peroxide from 45 to 196 mM had no significant effect on the initial droplet size. However, at concentrations of 386 mM and above, the initial droplet size increased, reaching 140 µm at a concentration of 1685 mM, which is 6.7 times larger than at 196 mM. This indicates that at high concentrations of hydrogen peroxide, the additional water introduced from the hydrogen peroxide solution affects emulsification by altering the phase distribution, leading to larger initial droplets. However, the percentage increase in droplet size after the reaction was not influenced by the concentration of hydrogen peroxide. For reactions oxidized by UHP, the initial droplet size was similar to that of aqueous hydrogen peroxide at concentrations of 196 and 385 mM. However, the emulsions were more stable against coalescence in UHP‐oxidized reactions, with a percentage increase in droplet size after the reaction of 12.2% and 70%, compared to 206% and 154% for hydrogen peroxide at concentrations of 196 and 386 mM, respectively. This increased stability is likely due to the controlled release of hydrogen peroxide by UHP, which mitigates its impact on interfacial tension and minimizes potential oxidative damage at the interface.

Figure 7e shows the influence of UHP and hydrogen peroxide concentrations in the influent solution on the synthesis of peroxyacetic acid. As expected, increasing the hydrogen peroxide concentration from 45 to 386 mM resulted in a corresponding increase in peroxyacetic acid concentration due to a shift in the reaction equilibrium toward the product (Table S2). However, further increasing the hydrogen peroxide concentration to 758 mM led to a decline in peroxyacetic acid concentration. This decrease is likely due to oxidative damage to the enzyme's active site or overall structure at such high peroxide concentrations, reducing its ability to efficiently interact with the substrate [58].

Consequently, reaction rates and catalytic productivity increased with oxidant concentrations up to 385 mM, but declined at higher concentrations (Table 1). The yield, however, decreased significantly from 83% to 5% as the hydrogen peroxide concentration increased from 45 to 1685 mM. Even at a high substrate concentration of 1685 mM, the enzyme was not completely deactivated, which is remarkable compared to other reaction media, such as the single organic phase, which has been reported to provide improved enzyme stability [3, 6]. However, in the single organic phase system reported by Meyer et al. complete enzyme deactivation occurred at a hydrogen peroxide concentration of 1680 mM before pseudo‐steady state was reached, despite the use of immobilized enzymes [6]. The maximum specific reaction rate at the optimal enzyme concentration (5 gL^−1^_dp_), achieved at 386 mM (17.5 mmolg^−^^1^ h^−^^1^) in this work is more than twice the rate observed in the single organic phase system, which was 6 mmolg^−^^1^h^−^^1^ at a comparable hydrogen peroxide concentration (384 mM) [6].

Although UHP provides a controlled release of anhydrous hydrogen peroxide, at a concentration of 196 mM, the yield and reaction rates were not significantly different from when hydrogen peroxide was used at the same concentration. However, at higher concentrations (385 mM), the product yield, and reaction rate were 15% lower when using UHP compared to hydrogen peroxide as shown in Table 1. This finding is consistent with the literature, in which Wiles et al. reported that hydrogen peroxide outperforms UHP at high concentrations [59]. They attributed this to the limited solubility of UHP in organic solvents such as ethyl acetate, which was used as the excess medium and continuous phase in this work. Therefore, for the integration of the second step in this process, the use of hydrogen peroxide as the oxidant is recommended.

## 4 Conclusions

This study demonstrates the effectiveness of PE as a sustainable medium for the first, enzymatic step of lipase catalyzed oxidative functionalization of alkenes in a continuous membrane reactor. Reaction rate, catalytic productivity, steady‐state yield, and emulsion stability were influenced by the pH and buffer concentration of the aqueous phase, with optimal performance achieved at a pH of 7 and a buffer concentration of 100 mM. Stability against coalescence and interfacial catalytic efficiency were further improved by modifying HDKH2000 nanoparticles through silanization and polymeric coatings. However, these modified nanoparticles had little impact on the yield and reaction rate.

Various operational parameters also significantly affected the reaction rate and product yield. Optimal conditions were identified at an enzyme concentration of 5 gL^−1^_dp_ and a temperature of 24°C, where a maximum product yield of 83% and a space‐time yield of 44.9 g_PA_L^−^^1^ d^−^^1^ were achieved at hydrogen peroxide concentrations of 45 and 386 mM in the influent solution, respectively. A specific reaction rate of 17.5 mmol g^−^^1^ h^−^^1^ was achieved at 386 mM hydrogen peroxide, more than twice the rate reported for single organic‐phase systems previously shown to enhance enzyme stability activity. Remarkably, even at a hydrogen peroxide concentration as high as 1685 mM, where immediate enzyme deactivation has been reported in such systems, lipase B in the PE system remained active beyond pseudo‐steady state, highlighting the protective role of the interfacial environment. This work highlights the potential of PE systems as robust, enzyme‐friendly platforms for high‐yield, continuous biocatalysis under demanding reaction conditions. The insights into interfacial catalysis, stability, and process optimization lay a foundation for integrating the subsequent step in the lipase‐catalyzed oxidative functionalization of alkenes as well as modeling the entire process. In addition, this work advances the scientific foundation for sustainable oxidative biotransformations, contributing to the development of scalable, greener routes for industrial oxidative functionalization.

## Nomenclature

A: Area, m^2^
C_Buffer_Buffer concentration, molL^−1^
: Buffer concentration, molL^−1^
C_CalB_
: Lipase B concentration, gL_dp_^−1^
C_oxidant_
: Oxidant concentration, molL^−1^
d_3,2_
: Sauter mean diameter, m
d_drop_
: Droplet diameter, m
J: Flux, Lm^−2^h^−1^
K: Partition coefficient, ‐
m: Mass, g
$\tilde{M}$
: Molar mass, gmol^−1^
N: Amount, mol
P: Productivity, gg^−1^h^−1^
ṙ: Specific reaction rate, molh^−1^g^−1^
Ṙ: Reaction rate, molL^−1^h^−1^
ṙ_A_
: Area‐specific reaction rate, molL^−1^h^−1^m^−2^
STY: Space‐time yield, gL^−1^d^−1^
t: Time, h
T: Temperature, ^0^C
V: Volume, L
X: Conversion, ‐
Y: Yield, ‐

### Greek symbols

φ: Volume phase fraction, ‐
τ: Residence time, h
ρ: Density, kgm^−3^

### Subscripts

aq: Aqueous phase
dp: Dispersed phase
eff: Effective
int: Interfacial
org: Organic phase
PA: Peroxyacetic acid

## Conflicts of Interest

The authors have declared no conflicts of interest.

## Supporting information




**Supporting File:** elsc70077‐sup‐0001‐SuppMat.docx.

## Data Availability

The data that support the findings of this study are available on request from the corresponding author.
