# Supplementary material for: Topic: Continuous Enzymatic Peracids Synthesis in Pickering Emulsions: Influence of Nanoparticles Modification, Aqueous Phase Composition and Operational Parameters
Source: Eng Life Sci. 2026 Apr 7;26(4):e70077. doi: 10.1002/elsc.70077 (PMC13054676; doi:10.1002/elsc.70077)
Supplement: Supplementary file 1 — Supporting File: elsc70077‐sup‐0001‐SuppMat.docx. [file ELSC-26-e70077-s001.docx]

**Supporting Information**

**Continuous enzymatic peracids synthesis in Pickering emulsions: Influence of nanoparticles modification, aqueous phase composition and operational parameters.**

Lemuel Onoriode Adomi^1^*, Sara Fatima Bhutta^2^, Marion Ansorge-Schumacher^2^ , Anja Drews^1^

^1^HTW Berlin – University of Applied Sciences, School of Life Science Engineering, Wilhelminenhofstraße 75A, 12459 Berlin, Germany

^2^Technische Universität Dresden, Institute of Microbiology, Chair of Molecular Biotechnology, Zellerscher weg 20b, 01217, Dresden, Germany

**Correspondence:** Lemuel Adomi, HTW Berlin – University of Applied Sciences, School of Life Science Engineering, Wilhelminenhofstraße 75A, 12459 Berlin, Germany, email: lemuel.adomi@htw-berlin.de

**Abstract**

The lipase-catalyzed oxidative functionalization of alkenes in Pickering emulsions (PE) offers a green and efficient alternative to conventional processes involving hazardous oxidants. Other investigated reaction media used for this alternative green pathway still suffer from enzyme deactivation and /or low specific reaction rate which limits their industrial adaptability. This study investigates the enzymatic synthesis of peroxyacetic acid, the first step in lipase-catalyzed oxidative functionalization, in a continuous membrane reactor. The effects of aqueous phase composition, modified silica nanoparticles, and operational parameters on reaction performance were systematically studied. Optimal conditions were obtained at pH 7, 100 mM buffer, and 5 g L_dp_⁻¹ enzyme concentration. Surface-modified silica nanoparticles improved PE stability and interfacial catalytic efficiency, while maintaining comparable catalytic productivity. Hydrogen peroxide outperformed urea hydrogen peroxide, yielding a maximum product yield of 83% at a concentration of 45 mM in the influent solution and a space-time yield of 44.9 g_PA_L⁻¹d⁻¹ at 386 mM. At 386 mM, the specific reaction rate (17.5 mmol g⁻¹ h⁻¹) was over twice that of reported single organic-phase systems. Despite the high peroxide concentrations, the enzyme displayed remarkable stability in PE due to the protective role of nanoparticles. This work provides critical insights into optimizing enzymatic oxidative functionalization in PE and their potential for sustainable industrial applications.

S1

**Table of contents**

1 Analytical procedure………………………………………………………………………………………………………………………S2

1.1 Determination of effluent concentration…………………………………..…………………………………………..…..S2

2 Controller Performance……………….………………………………………………………………………………………………..S2

3 Concentration of substrate and Peroxyacetic acid at different reaction conditions**……………….**………S3

**1 Analytical procedure**

**1.1 Determination of effluent concentration**

The peracetic acids and hydrogen peroxide or urea hydrogen peroxide concentrations were analyzed simultaneously with the HPLC, by a derivatization reaction as stated in section 2.8. The HPLC hence analyzed the concentration of the methyl p-tolyl sulfoxide (MTSO) and triphenylphosphine oxide (TPPO) as that of the peracetic acid and hydrogen peroxide respectively. Samples were diluted to the desired measurement range as stated in section 2.8 and shown in Fig. S1. Afterwards, to 300 µL of water, 100 µL of 20 mM methyl p-tolyl sulfide (MTS) and 100 µL of diluted sample or blank (ethyl acetate) was added. The mixture was incubated for 20 mins. 300 µL acetonitrile and 200 µL of 10 mM triphenylphosphine (TPP) was then added to the mixture and incubated in the dark for 30 minutes. The retention times for MTSO, TPPO, MTS and TPP were 0.95 min, 1.45 min, 3.88 min and 15.36 min respectively.

a

**Figure S1.** Calibration curve for a) MTSO and b) TPPO analysis

**2 Controller Performance**

The integrated flux control performance in the LabVIEW program was evaluated by analyzing the deviation between the actual flux and set flux as well as the time required to achieve steady state. The PID parameters were tuned using an open loop method. The resulting tuned PID values were K_p_ = 4 bar.m^2^hL^-1^, T_i_ = 36.7 sec and T_d_ = 8.9 sec.

S2

To evaluate the controller's accuracy, the reactor was filled with Pickering emulsion (φ = 0.3, C_particles_ = 3 %wt/wt_dp_, C_CalB_ = 5 gL^-1^_dp_, C_H2O2_ = 100mM), and the feed solution was ethyl acetate saturated with 100mM hydrogen peroxide, replicating the composition used in a reaction experiment. As shown in Fig. S2, steady state was achieved within 4 mins, indicating the controller's ability to rapidly stabilize the system. Additionally, for various set flux values (10 – 30 Lm^-2^h^-1^), the steady-state error was consistently below 5% (Table S1). The actual values presented in Table S1 represent the average actual flux after steady-state was achieved.

**Table S1:** Steady state error at different set points.

| Set Point | Actual value | Error |
| --- | --- | --- |
| Lm^-2^h^-1^ | | % |
| 10 | 9.57 | 4.34 |
| 15 | 14.39 | 4.05 |
| 20 | 19.20 | 3.98 |
| 25 | 24.06 | 3.74 |
| 30 | 28.94 | 3.53 |

**Figure S2.** Performance of the flux control system at different flux values. T = 24 ^O^C, n = 500 min^-1^

**3 Concentration of substrate and Peroxyacetic acid at different reaction conditions**

Table S2 below shows the concentration of the product and reactant at different reaction conditions and PE composition at steady state conditions. In addition, the product selectivity(S) is shown.

S3

**Table S2:** Composition of PE and operational parameters influence on the synthesis of peroxyacetic acid. C_particles_ = 3 %wt/wt_dp_, φ = 0.3.

| C_buffer_ | pH | Nanoparticles | C_CalB_ | T | τ | C_oxidant,in_ | C_PA_ | C_Oxidant_, | S |
| --- | --- | --- | --- | --- | --- | --- | --- | --- | --- |
| [mM] | [-] | [-] | [gL^-1^_dp_] | [^0^C] | [h] | [mM] | [mM] | [mM] | [%] |
| 10  50  100  300 | 7 | unmodified | 1 | 30 | 4.6 | 106 | 33.9 | 62.4 | 0.77 |
|  |  |  |  |  |  |  | 33.6 | 64.4 | 0.80 |
|  |  |  |  |  |  |  | 37.1 | 63.6 | 0.86 |
|  |  |  |  |  |  |  | 31.2 | 69.8 | 0.85 |
| 100 | 5 | unmodified | 5 | 24 | 4.6 | 153 | 80.9 | 60.8 | 0.88 |
|  | 6 |  |  |  |  |  | 88.6 | 48.4 | 0.85 |
|  | 7 |  |  |  |  |  | 94.8 | 49.9 | 0.92 |
|  | 8 |  |  |  |  |  | 94.8 | 47.0 | 0.90 |
|  | 9 |  |  |  |  |  | 91.3 | 50.8 | 0.90 |
|  | 10 |  |  |  |  |  | 88.4 | 55.5 | 0.91 |
| 50 | 7 | unmodified | 1 | 30 | 4.6 | 289 | 100.8 | 166.5 | 0.82 |
|  |  | TMOD |  |  |  |  | 91.1 | 176.5 | 0.81 |
|  |  | Amp |  |  |  |  | 95.1 | 179.3 | 0.86 |
|  |  | Phil |  |  |  |  | 91.8 | 197.6 | 1.00 |
|  |  | Phob |  |  |  |  | 92.2 | 188.8 | 0.92 |
| 100 | 7 | unmodified | 1 | 24 | 6.9 | 106 | 46.4 | 52.8 | 0.88 |
|  |  |  | 3 |  |  |  | 71.2 | 30.1 | 0.94 |
|  |  |  | 5 |  |  |  | 76.2 | 28.4 | 0.98 |
|  |  |  | 10 |  |  |  | 76.3 | 28.4 | 0.99 |
| 100 | 7 | unmodified | 1 | 24 | 4.6 | 106 | 37.0 | 67.6 | 0.95 |
|  |  |  |  | 30 |  |  | 37.1 | 63.6 | 0.86 |
|  |  |  |  | 40 |  |  | 20.1 | 81.5 | 0.80 |
| 100 | 7 | unmodified | 5 | 24 | 14.7 | 106 | 85.2 | 17.4 | 0.96 |
|  |  |  |  |  | 10.1 |  | 81.9 | 20.0 | 0.95 |
|  |  |  |  |  | 6.9 |  | 76.2 | 28.4 | 0.98 |
|  |  |  |  |  | 4.7 |  | 67.7 | 28.8 | 0.88 |
|  |  |  |  |  | 3.8 |  | 66.1 | 23.9 | 0.81 |
|  |  |  |  |  | 3.4 |  | 62.6 | 24.1 | 0.77 |
| 100 | 7 | unmodified | 5 | 24 | 6.9 | 45 | 37.3 | 7.2 | 0.99 |
|  |  |  |  |  |  | 106 | 76.2 | 28.4 | 0.98 |
|  |  |  |  |  |  | 196 | 128.1 | 54.8 | 0.91 |
|  |  |  |  |  |  | 386 | 169.2 | 162.5 | 0.76 |
|  |  |  |  |  |  | 758 | 147.6 | 548.9 | 0.71 |
|  |  |  |  |  |  | 1685 | 85.1 | 1452.9 | 0.37 |
| 100 | 7 | unmodified | 5 | 24 | 6.9 | 196 | 127.1 | 46.2 | 0.85 |
|  |  |  |  |  |  | 386 | 144.3 | 164.2 | 0.65 |

S4
